# Syndrome de régression embryonnaire des testicules: à propos de 6 cas

**DOI:** 10.11604/pamj.2014.18.250.3819

**Published:** 2014-07-26

**Authors:** Hanane Latrech, Mohammed El Hassan Gharbi, Abdelmjid Chraïbi, Ahmed Gaouzi

**Affiliations:** 1 Department of Endocrinology, Medical school, Mohamed First University, Oujda, Morocco; 2 Department of Endocrinology, Medical school, Mohammed V Souissi University, Ibn Sina hospital, Rabat, Morocco; 3 Department of Pediatrics, Medical school, Mohammed V Souissi University, Rabat, Morocco

**Keywords:** Régression testiculaire, génétique, AMH, HCG, cœlioscopie, testicular regression, genetic, AMH, HCG, coelioscopy

## Abstract

Le syndrome de régression embryonnaire des testicules ou anorchidie bilatérale congénitale (ABC) est un syndrome très rare défini par l'absence complète de tissu testiculaire chez un patient présentant un caryotype masculin normal. Le phénotype est variable en fonction du moment où la régression gonadique survient in utero. Actuellement, son déterminisme reste encore mystérieux mais sa survenue familiale est un argument pour suggérer une étiologie génétique. Nous en rapportons six cas, illustrant la variabilité phénotypique et décrivant la démarche et les nouveaux marqueurs diagnostiques ainsi que la conduite à tenir thérapeutique.

## Introduction

Le syndrome de régression embryonnaire des testicules (SRET) ou anorchidie bilatérale congénitale (ABC) est défini par l′absence complète de tissu testiculaire chez un patient présentant un caryotype mâle normal [1–3]. Sa prévalence est, approximativement, de 1 cas par 20 000 mâles mais reste souvent sous-estimée et prise, souvent cliniquement, à tort pour une cryptorchidie bilatérale de siège intra-abdominal [2]. Le phénotype est variable en fonction du moment où la régression gonadique survient in utéro. Son étiologie est encore discutée mais sa survenue familiale est un argument pour suggérer une étiologie génétique [2]. La démarche diagnostique de cette entité nécessite des investigations paracliniques voire une coelioscopie. Nous rapportons six cas d'ABC, illustrant la variabilité phénotypique, à travers lesquels nous décrivons la démarche diagnostique tout en discutant les nouveaux marqueurs diagnostiques et la conduite à tenir thérapeutique.

## Méthodes

De 1999 à 2009, nous avons reçu six patients en consultation dans l'unité d'endocrinologie pédiatrique de Rabat pour une suspicion d'anorchidie bilatérale. Les paramètres cliniques étudiés sont l’âge du diagnostic, l’état des organes génitaux externes (en appréciant la longueur, la largeur, le nombre d'orifices, données de la palpation), un éventuel syndrome dysmorphique associé et la présence de cas similaires dans la famille. L'exploration hormonale a comporté le dosage de l'hormone anti- Müllerienne (AMH) (EIA Immunotech), de la testostérone de base et après stimulation par HCG (Human ChorioGonadotropin) (6 injections intra- musculaires de 1500 UI d'HCG toutes les 48 heures avec réalisation d'un prélèvement le lendemain de la dernière injection pour dosage de la testostérone plasmatique). La réalisation du caryotype sur du sang périphérique était systématique. L'exploration morphologique a comporté la réalisation d'une échographie abdominale +/- une tomodensitométrie abdomino-pelvienne. L'exploration coelioscopique a toujours été préconisée.

## Résultats

**Figure 1:**
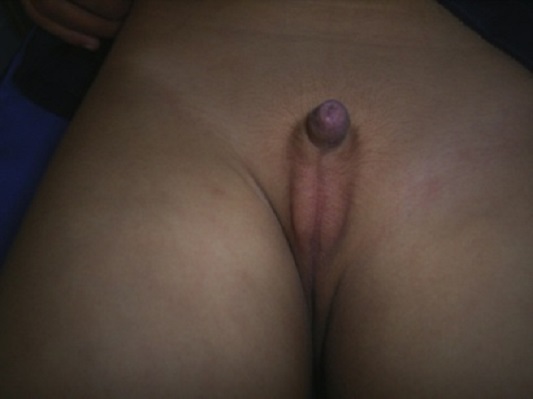
Micropénis avec bourses vides (1^ère^ observation)

L’âge moyen du diagnostic était de 4,6 ans avec des extrêmes de 16 mois à 11 ans. Deux de nos patients avaient un antécédent familial de cryptorchidie. Le micropénis était présent dans deux cas (Figure 1). La testostéronémie était très basse dans tous les cas et ne répondait pas à la stimulation par l'HCG. Ce test n'a pas été réalisé dans un cas. Le dosage de l'AMH, réalisé chez cinq patients, était toujours très bas. L'exploration coelioscopique, réalisée dans cinq cas (non réalisée chez le 6^ème^ patient du fait du refus de la famille), a permis de confirmer le diagnostic d'ABC. Le Tableau 1 résume les données cliniques et paracliniques de nos patients.

**Tableau 1:** Paramètres étudiés à propos de 6 observations d'anorchidie

Patients	Age	Phénotype	Caryotype	AMH (ng/ml)	TNE de base (ng/ml)	Après HCG (ng/ml)	Echographie /TDM	Cœlioscopie
**1**	6 ans	Microppénis (2 cm/0.5 Pas de gonade palpable	46 XY	0.7	< 0.1	0.2	Pas de gonade	Faite
**2**	28 mois	Verge normale Pas de gonade palpable	46 XY	0.1	<0.1	0.28	Pas de gonade	Faite
**3**	16 mois	Micropénis (1.7 cm/0.5) Pas de gonade palpable	46 XY	0.008	0.02	NF	Pas de gonade	Faite
**4**	4 ans	Verge normale Pas de gonade palpable	46 XY	0.3	Indetectable	<0.1	Pas de gonade	Faite
**5**	11 ans	Verge normale Pas de gonade palpable	46 XY	NF	0.01	0.05	Pas de gonade (TDM)	Faite
**6**	3 ans et 6 mois	Verge limite (3/2 cm) Pas de gonade palpable	46 XY	0.2	Indétectable	<0.5	Pas de gonade	NF

NF : non fait (not done)

## Discussion

**Figure 2:**
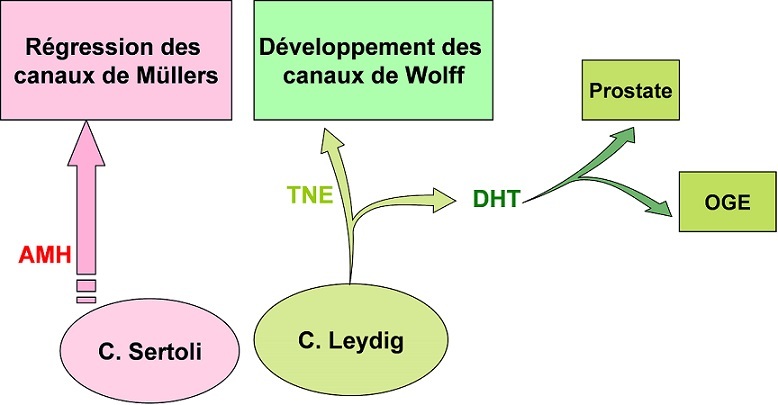
Différenciation des organes génitaux externes et internes masculins

Le syndrome de régression embryonnaire des testicules est défini par une absence, partielle ou complète, du tissu testiculaire en présence d'un caryotype 46 XY [1–3]. Ce syndrome fait partie du large spectre clinique des dysgénésies gonadiques partielles 46 XY. La plupart des patients atteints présentent une anomalie de la différenciation sexuelle ou un micropénis avec une régression complète du tissu testiculaire qui peut être uni- ou bilatérale. Le degré de masculinisation des organes génitaux externes et internes dépend de la durée du fonctionnement testiculaire avant son affaissement expliquant la variabilité phénotypique [4, 5]. En effet, en présence d'organes génitaux externes masculins normaux, comme cela était le cas chez trois de nos patients, on peut supposer que le testicule foetal était bien présent et fonctionnel durant la période précoce du développement avec une production suffisante d'androgènes, pour assurer un développement normal ou subnormal des organes génitaux externes mâles, et aussi une sécrétion adéquate d'hormone anti-Mullerienne, permettant une régression des dérivés mülleriens (Figure 2). Ceci sous- entend que, dans cette condition, la régression gonadique est survenue tardivement dans la vie foetale, au-delà de la 12ème semaine de grossesse [2, 5, 6–8].

Cependant, devant l'association d'un micropénis, rapporté chez trois de nos patients, et d'une anomalie du développement des canaux sexuels souvent retrouvée, certains auteurs ont suggéré la présence préalable d'une altération intrinsèque du tissu testiculaire avant sa régression [4, 8, 9]. Un phénotype féminin avec ou sans ambiguïté des organes génitaux externes, une absence de gonade avec présence d'un utérus hypoplasique est possible suggérant une régression testiculaire précoce, entre 8 et 12 semaines de grossesse. Cette forme pose au clinicien un problème d'orientation du sexe. Le SRET, situation assez bien connue par les urologues et les chirurgiens pédiatres, affecte approximativement 5% des enfants cryptorchides [9, 10] ce qui rend compte de la sous estimation de ce diagnostic qui n'est pas si rare qu'on le pense. Dans notre expérience, nous avons recensé six cas d'ABC parmi 120 patients admis pour des anomalies du développement sexuel (soit 5%). Plusieurs mécanismes ont été évoqués pour expliquer ce phénomène. Certains ont pensé à un mécanisme de torsion en période foetale ou périnatale devant la mise en évidence de macrophages haemosiderin-laden sur les pièces opératoires. Les traumatismes ou les occlusions vasculaires sont aussi possibles [2, 3, 9, 11, 12].

Finalement, une étiologie génétique possible a été envisagée sur la base d′une survenue familiale. En effet, la description de familles consanguines comprenant plusieurs sujets atteints laisse penser à une transmission autosomique récessive. En plus, plusieurs équipes ont évoqué l′existence d′anomalies au niveau des gènes impliqués dans le développement ou la descente des gonades (SRY, INSL3 or LGR8) mais aucune mutation n'a été documentée en cas d'anorchidie [2, 9, 13]. Partant du principe que le Steroidogenic Factor 1 (SF1/AdBP4/FTZF1, NR5A1) est connu pour son rôle crucial dans le développement des glandes surrénales et des testicules aussi bien que dans la régulation de la stéroïdogenèse et la reproduction, une étude française a pu mettre en évidence une nouvelle mutation partielle hétérozygote (V355M) du SF1 chez un garçon présentant un SRET et un micropénis [2]. Sur le plan biologique, le dosage de l'hormone anti-mullerienne (AHM) comme étant un marqueur de la présence du tissu testiculaire, est extrêmement bas ou indétectable avec une absence de réponse de la testostérone plasmatique à la stimulation par l'HCG [2, 10, 11, 14, 15]. Misra et al [16] ont rapporté une spécificité et une valeur prédictive positive plus élevée de l'AMH par rapport au test à l'HCG pour le diagnostic. L'association d'un taux d'AMH indétectable et des gonadotrophines élevées, en période pubertaire, est très vraisemblablement en faveur d'un SRET. Chez les enfants pré pubères, dont le taux de gonadotrophines n'est pas élevé, le test de stimulation à l'HCG s'avère donc nécessaire pour valider le diagnostic d'une anorchidie et aussi pour éliminer les rares cas de persistance des canaux de Müller ou des erreurs de dosages.

En effet, la valeur d'AMH pourrait être faussement basse en cas d'expédition du sérum à une température ambiante supérieure à 4°C ou congelé. En plus, au cours de cette période pré- pubertaire, les cellules de Leydig sont réfractaires à l'HCG d'où l'intérêt pour certains d'un test de stimulation prolongée pendant plusieurs semaines afin d'induire une production adéquate d'androgènes [16]. Chez un de nos patients, dont le test de stimulation n'a pas été réalisé, nous nous sommes contentés du taux indétectable de l'AMH pour indiquer une exploration coelioscopique qui a, par la suite, confirmé le diagnostic. Dans les autres cas, nous avons noté une parfaite concordance entre les résultats de l'AMH et le test de stimulation à l'HCG. L’échographie, en plus de son caractère opérateur dépendant, ainsi que l'imagerie par résonnance magnétique peuvent parfois ne pas mettre en évidence de tissu testiculaire [17, 18]. La confirmation diagnostique ne peut être apportée que par l'exploration coelioscopique et dont le recours reste controversé [3, 9, 10, 19–21]. Lorsqu'elle est réalisée, elle confirme l'absence de gonade ou la présence de testicules rudimentaires, mais aussi l'existence d'une disposition normale du pédicule spermatique et du canal déférent témoignant d'une migration normale du testicule qui s'est trouvé détruit tardivement dans la vie foetale [19]. En plus, la confirmation histologique de la régression testiculaire rassure, généralement, le chirurgien pour ce qui est de sa démarche diagnostique mais aussi la famille en mettant fin aux investigations paracliniques [9, 10]. Plusieurs aspects anatomopathologiques peuvent être retrouvés en proportions variables: calcifications, hémosidérines, nodules, cordons spermatiques et épididymes [3, 9, 12]. L'examen anatomopathologique des pièces opératoires n'a permis d'identifier du tissu testiculaire que dans 10% des cas sans présence de cellules germinales. Ceci pourrait plaider en faveur d'un risque négligeable de dégénérescence maligne et donc d'une exploration coelioscopique non systématique [3, 22, 23].

D'autre part, certains auteurs ont rapporté parfois la présence de cellules germinales sur des pièces opératoires dans 11% des cas [9, 24] et de tubes séminifères dans 0 à 40% [10, 24–27]. Ces éléments ont été considérés comme viables dans 0 à 16% des cas faisant craindre un risque potentiel de dégénérescence maligne. L'issue ultérieure de ces éléments résiduels n'est pas encore connue [9, 20, 24, 28]. De ce fait, nous pensons que l'exploration coelioscopique, réalisée chez cinq de nos patients, garde encore toute sa place et doit être toujours indiquée permettant ainsi de conclure définitivement sur le statut des gonades. L'androgénothérapie substitutive et l'implantation de prothèses restent, malheureusement, les seules possibilités thérapeutiques à proposer à ces patients [14]. L'association d'un micropénis chez trois de nos patients, a nécessité une androgénothérapie instaurée le plutôt possible afin d’éviter d'alourdir encore plus leur vécu psychologique à l'adolescence et à l’âge adulte. L'implantation de prothèses testiculaires peut être envisagée pour des raisons psycho sociales et esthétiques [29] mais à réaliser avant le début du traitement substitutif. Cette technique peut être grevée de complications à type d'inflammation ou de perforation de la peau.

## Conclusion

Le SRET est retrouvé chez 5% des enfants cryptorchides. Plusieurs hypothèses ont été évoquées. Une origine génétique, longtemps suspectée, a été documentée devant la présence de mutation au niveau du gène de la SF1. Un taux d'AMH indétectable associé à une absence de réponse de la testostérone plasmatique à la stimulation par l'HCG fait le diagnostic d'une anorchidie. L'exploration coelioscopique reste le seul moyen de confirmation diagnostique permettant au médecin de conclure définitivement sur le statut de la gonade et d’éviter un risque, même minime, de dégénérescence maligne. Les possibilités thérapeutiques se limitent actuellement à une androgénothérapie substitutive qui pourrait être associée à une implantation de prothèses. Une prise en charge psychologique initialement des parents puis du patient lui-même est nécessaire pouvant ainsi améliorer le vécu de la situation à un âge plus grand.
